# Standardized Ileal Digestibility of Protein and Amino Acids in Black Soldier Fly Larvae and Duckweed in Broiler Chickens

**DOI:** 10.3390/ani16030461

**Published:** 2026-02-01

**Authors:** Chanwit Kaewtapee, Hathaipat Thongthung, Krittaya Petchpoung, Masaaki Morikawa, Sirinapa Chungopast

**Affiliations:** 1 Department of Animal Sciences, Faculty of Agriculture, Kasetsart University, Bangkok 10900, Thailand; hathaipat.thong@ku.th; 2 Duckweed Holobiont Resource & Research Center (DHbRC), Kasetsart University, Bangkok 10900, Thailand; morikawa@ees.hokudai.ac.jp (M.M.); agrsrnp@ku.ac.th (S.C.); 3 Scientific Equipment and Research Division, Kasetsart University Research and Development Institute, Kasetsart University, Bangkok 10900, Thailand; rdikyp@ku.ac.th; 4 Graduate School of Environmental Science, Hokkaido University, Sapporo 060-0810, Hokkaido, Japan; 5 Department of Soil Science, Faculty of Agriculture at Kamphaeng Saen, Kasetsart University Kamphaeng Saen Campus, Nakhon Pathom 73140, Thailand

**Keywords:** alternative feed ingredients, aquatic plants, insect meal, poultry nutrition

## Abstract

Black soldier fly larvae (BSFL) and duckweed are potential alternative feed ingredients for sustainable poultry production. This study aimed to determine the standardized ileal digestibility (SID) of amino acids (AAs) in BSFL, *Lemna*, and *Spirodela* compared with soybean meal and rapeseed meal. Six cages of eighteen broiler chickens were allocated to each treatment. The crude protein (CP) content was highest in soybean meal (511 g/kg dry matter; DM), intermediate in BSFL (391 g/kg), and rapeseed meal (335 g/kg DM) and lowest in *Lemna* (185 g/kg DM) and *Spirodela* (145 g/kg DM). Ether extract was highest in BSFL (95 g/kg DM), whereas crude fiber was highest in *Lemna* (109 g/kg DM) and *Spirodela* (171 g/kg DM). The SID of CP was higher (*p* < 0.05) in BSFL (89.0%) than in soybean meal (82.3%), rapeseed meal (71.3%), *Lemna* (70.2%), and *Spirodela* (44.9%). The SID of all essential amino acids (AAs) was higher (*p* < 0.05) in *Lemna* than in *Spirodela*, but it did not differ from that in rapeseed meal. In conclusion, the high fat content in BSFL can enhance AA digestibility, whereas the use of duckweed may be limited by its fiber fractions, which negatively impact the SID of CP and AAs in broiler chickens.

## Simple Summary

Insect meal and duckweed are increasingly recognized as novel feed ingredients that can contribute to sustainable poultry production. Determining the standardized ileal digestibility of crude protein and amino acids is essential for formulating nutritionally balanced diets and evaluating the potential inclusion of insect meal and duckweed in broiler chicken nutrition. Black soldier fly is an insect characterized by rapid growth, a short rearing cycle, and the ability to utilize a wide range of substrates. The high fat content in black soldier fly larvae resulted in greater amino acid digestibility than soybean meal and rapeseed meal. Duckweed, including *Lemna* and *Spirodela*, is a tiny aquatic floating plant that represents a valuable source of plant protein. However, its high fiber, tannin, and ash contents can reduce amino acid digestibility. Therefore, black soldier fly larvae and duckweed can be supplemented in poultry diets, but differences in chemical composition and amino acid digestibility should be carefully considered during feed formulation to meet the nutritional requirements and effectively optimize the growth performance of broiler chickens.

## 1. Introduction

In poultry production, feed costs represent the primary component of total production costs, driving increasing interest in sustainable alternatives to conventional soybean-based feed ingredients. In this regard, black soldier fly larvae (BSFL; *Hermetia illucens*; [1,2]) and duckweed [3] are considered potential feed ingredients for sustainable poultry production. Black soldier fly larvae have emerged as a highly promising feed ingredient for broiler chicken diets due to their rich protein content and essential amino acids (AAs; [1]). The production of BSFL requires minimal water and land resources compared to traditional livestock feed and can utilize organic waste, contributing to waste reduction and nutrient recycling [4,5]. Recent research has indicated that BSFL can be included in broiler chicken diets at levels up to 20% without negatively affecting growth performance or the feed efficiency ratio [6]. Furthermore, the presence of chitin in BSFL may enhance immune function [7] and inhibit pathogenic bacteria in host animals [8]. Therefore, the inclusion of BSFL in diets aligns with circular economy principles, promoting sustainability and resource efficiency in poultry meat production.

Duckweed is a tiny, floating, and fast-growing aquatic plant belonging to the Lemnaceae family, which comprises five genera: *Spirodela*, *Landoltia, Lemna*, *Wolffiella*, and *Wolffia* [9]. It has a high growth rate, doubling in amount within only 2 to 7 days, resulting in rapid biomass production [10]. Duckweed is an abundant source of protein, AAs, carbohydrates, and pigments (beta-carotene and xanthophyll), which can be supplemented in the diets of poultry and aquatic animals [11]. A recent study by Demann et al. [12] reported that the standardized ileal digestibility (SID) of AAs in diets containing *Lemna* or *Spirodela* ranged from 40.2 to 83.7%, and *Lemna* can be included in broiler chicken diets at levels up to 25% depending on its AA content and digestibility. Furthermore, the supplementation of 6% duckweed as a replacement for sesame oil cake in broiler chicken diets has been shown to improve body weight, feed intake, and the feed conversion ratio [13]. This information indicates that duckweed can serve as a viable alternative protein source in poultry diets.

Understanding the digestibility of feed ingredients is essential for formulating nutritionally balanced diets and evaluating the inclusion of alternative protein sources in poultry nutrition [14]. SID values provide accurate estimates of AA bioavailability, allowing for precise diet formulation that meets the nutritional requirements and optimizes nutrient utilization [15]. However, research on the SID of AAs in BSFL and duckweed is limited compared to that on commonly used protein sources such as soybean meal and rapeseed meal, and determining the SID of AAs in these alternative ingredients is essential for optimizing their potential as sustainable feed ingredients in poultry diets. It was hypothesized that BSFL and duckweed exhibit different SID of protein and AAs due to variations in their chemical composition. Therefore, the objective of this study was to determine the SID of AAs in BSFL and duckweed for broiler chickens.

## 2. Materials and Methods

The research protocol was reviewed and approved by the Animal Care and Use for Scientific Research Committee, Kasetsart University, Bangkok, Thailand (ACKU68-KULAC-005), and animals were cared for in accordance with the ethical principles for the use of animals for scientific purposes [16].

### 2.1. Birds and Management

A total of ninety one-day-old Cobb 500 male broiler chickens were raised in floor pens. All birds were fed a commercial diet (230 g/kg crude protein; CP) until day 28. Feed and water were provided ad libitum. The temperature in the house was controlled at 32 °C during the first week and gradually decreased to 25 °C during the second week, where it was maintained until the end of the experiment. On day 28, three birds were individually weighed and randomly allotted to 30 cages (50 cm width × 55 cm length × 65 cm height), each equipped with a feeding cup and a nipple drinker.

### 2.2. Experimental Design and Assay Diets

BSFL were obtained from the Department of Entomology, Faculty of Agriculture, Kasetsart University (Bangkok, Thailand). The BSFL were starved for 4 h and subsequently inactivated by freezing at −20 °C. Thereafter, the BSFL were dried using a microwave oven (EMM2023MW, Electrolux Co., Ltd., Stockholm, Sweden) at 500 W for 10–12 min. *Lemna* (*Lemna aequinoctialis*) and *Spirodela* (*Spirodela polyrhiza*) were provided by Advanced Greenfarm Co., Ltd. (Nakhon Pathom, Thailand). *Lemna* and *Spirodela* were harvested from cultivation ponds, washed with clean water, and dried at 50–55 °C for 72 h in a hot-air oven (Model ED 115, Binder GmbH, Tuttlingen, Germany). Soybean meal was purchased as a commercial product from Thai Vegetable Oil Public Co., Ltd. (Bangkok, Thailand), whereas rapeseed meal was obtained from Sun Feed Co., Ltd., (Bangkok, Thailand). The analyzed chemical composition of the test ingredients is presented in Table 1.

This study examined six treatments with six replications in a completely randomized design, with each cage containing three birds considered as the experimental unit. Five assay diets, including soybean meal, rapeseed meal, BSFL, *Lemna*, and *Spirodela*, were formulated (Table 2) to meet or exceed the nutrient requirements recommended by Cobb 500 (Cobb-Vantress, Inc., Siloam Springs, AR, USA). Basal endogenous AA losses were determined using a nitrogen-free diet (Table 3), following the formulation described by Adedokun et al. [17] to minimize the variability in SID estimation [15]. Chromic oxide was supplemented at a level of 0.3% in the assay diets and 0.5% in the nitrogen-free diet as an indigestible indicator, in accordance with Ravindran et al. [15].

On day 35, all birds were euthanized via asphyxiation with carbon dioxide; the body cavity of each bird was immediately opened, and the ileal content was collected. The ileum was defined as the section between Meckel’s diverticulum and 2 cm anterior to the ileo–ceco–colonic junction. The ileal contents were gently flushed out with distilled water, pooled from three birds from one cage, and immediately frozen. Subsequently, the samples were freeze-dried and finely ground to 0.5 mm before chemical analysis.

### 2.3. Chemical Analysis

A proximate analysis was conducted according to the official standard procedure [18]. Dry matter (DM) was determined using a hot-air oven (UF, Memmert GmbH + Co. KG, Schwabach, Germany) at 103 to 105 °C for 4 to 6 h (method 930.15). Nitrogen (N) was measured using the Kjeldahl procedure, and the CP content was calculated as N × 6.25 (method 984.13). The ether extract (EE) was determined using Soxhlet extraction (method 920.38). Crude fiber (CF) was obtained following digestion in sulfuric acid and sodium hydroxide (method 978.10). Neutral detergent fiber (NDF) was determined using a neutral detergent solution (method 2002.04), and acid detergent fiber (ADF) was determined using an acid detergent solution (method 973.18). The ash content was determined via incineration in a muffle furnace at 600 °C for 6 h (method 942.05). The tannin content was analyzed following the Folin–Ciocalteu method [19]. The chromium contents in the assay diets and ileal digesta samples were analyzed using a UV spectrophotometer (SP-UV1100, DLAB Scientific Co., Ltd., Beijing, China) according to the standard procedure (method 968.088D).

The AA contents in the feed ingredients and assay diets and the ileal contents were analyzed by Global Facility Center, Hokkaido University, using an amino acid analyzer (Hitachi L-8900, Hitachi High-Technologies Corporation, Tokyo, Japan). The samples were hydrolyzed with 6 N HCl containing 0.1% phenol at 110 °C for 24 h in an oven. For sulfur-containing AAs, cystine and methionine were analyzed as cysteic acid and methionine sulphone, respectively, following oxidation with performic acid prior to acid hydrolysis. Tryptophan was not determined. The internal standard nor-Leucine (143-02781, Fujifilm Wako Pure Chemical Industries, Ltd., Tokyo, Japan) was added to the mixture. Thereafter, the samples were centrifuged and filtered to pass through a 0.22 µm syringe filter. The AA profile was measured with ninhydrin post-column derivatization (Wako Pure Chemical Industries, Ltd., Osaka, Japan) and separated using an ion-exchange column (4.6 mm ID × 80 mm length; 2620 MPH, Hitachi High-Technologies Corporation, Tokyo, Japan) at a flow rate of 0.19 mL/min. The chromatograms detected with a photometric detector (UV–Visible) at 570 and 440 nm were integrated using EZChrom Elite software, version 3.3.2 (Agilent Technologies Inc., Santa Clara, CA, USA).

### 2.4. Calculation and Statistical Analyses

The apparent ileal digestibility (AID) of AAs was calculated using chromic oxide (Cr) as the indigestible marker as follows:

AID (%) = 100 − {[(AA/Cr)i/(AA/Cr)d] × 100}

Here, (AA/Cr)d is the ratio of AA to chromic oxide in the diet, and (AA/Cr)i is the ratio of AA to chromic oxide in the ileal digesta.

The SID was calculated from AID using the basal endogenous AA losses determined following the feeding of a nitrogen-free diet.

SID (%) = AID + {[Basal EAA (g/kg DM intake)]/[Ingredient AA (g/kg DM intake)]}

Data were analyzed statistically via an analysis of variance (ANOVA) according to a completely randomized design. The experimental unit was the cage. Statistical analyses were performed using the GLM procedure of SAS^®^ OnDemand for Academics, based on SAS version 9.4 (SAS Institute Inc., Cary, NC, USA). When a significant treatment effect was detected, mean differences among treatments were separated using Tukey’s multiple comparison test. Differences were considered statistically significant at *p* < 0.05.

## 3. Results

As shown in Table 1, the CP content was higher in soybean meal (511 g/kg DM) than in *Lemna* (185 g/kg DM) and *Spirodela* (145 g/kg DM), with intermediate levels in BSFL (391 g/kg DM) and rapeseed meal (335 g/kg DM). The EE content was higher in BSFL (95 g/kg DM) and lower in rapeseed meal (14 g/kg DM) and soybean meal (12 g/kg DM). The ash content was higher in *Spirodela* (212 g/kg DM) and *Lemna* (142 g/kg DM) than in BSFL (112 g/kg DM), rapeseed meal (109 g/kg DM), and soybean meal (72 g/kg DM). The CF content was higher in *Spirodela* (171 g/kg DM) and *Lemna* (109 g/kg DM) than in soybean meal (30 g/kg DM). The NDF content was higher in *Spirodela* (338 g/kg DM), *Lemna* (316 g/kg DM), and rapeseed meal (269 g/kg DM) than in BSFL (165 g/kg DM) and soybean meal (107 g/kg DM). Likewise, the ADF content was higher in *Spirodela* (244 g/kg DM), *Lemna* (185 g/kg DM), and rapeseed meal (187 g/kg DM) than in BSFL (90 g/kg DM) and soybean meal (68 g/kg DM). The tannin content was highest in *Spirodela* (14.1 mg/g) and lowest in soybean meal (1.3 mg/g), with intermediate levels in rapeseed meal (5.7 g/kg DM) and *Lemna* (5.5 g/kg DM). The contents of almost all essential AAs were higher in soybean meal, ranging from 6.8 g/kg DM for methionine to 41.1 g/kg DM for leucine, than in rapeseed meal, ranging from 7.1 g/kg DM for methionine to 26.0 g/kg DM for leucine. With regard to BSFL and duckweed, the contents of almost all essential AAs were higher in BSFL, ranging from 6.4 g/kg DM for methionine to 29.0 g/kg DM for leucine, than in *Lemna*, ranging from 3.5 g/kg DM for methionine to 16.4 g/kg DM for leucine, and *Spirodela*, ranging from 2.5 g/kg DM for methionine to 11.8 g/kg DM for leucine.

The AID and SID of the crude protein and AAs of soybean meal, rapeseed meal, BSFL, *Lemna*, and *Spirodela* are presented in Table 4 and Table 5, respectively. The AID of CP was higher (*p* < 0.05) in BSFL (82.5%) and soybean meal (75.8%) than in rapeseed meal (64.8%) and *Lemna* (60.8%), whereas the lowest SID of CP was observed in *Spirodela* (35.5%). Likewise, the SID of CP was higher (*p* < 0.05) in BSFL (89.0%) and soybean meal (82.3%) than in rapeseed meal (71.3%) and *Lemna* (70.2%), whereas the lowest SID of CP was observed in *Spirodela* (44.9%). The SID of all essential AAs was higher (*p* < 0.05) in BSFL, ranging from 90.0% for histidine to 96% for threonine, than in soybean meal, ranging from 79.2% for valine to 85.0% for arginine, and rapeseed meal, ranging from 70.6% for isoleucine to 81.6% for arginine. The SID values of all essential AAs in *Lemna*, ranging from 70.6% for isoleucine to 80.1% for arginine, were higher (*p* < 0.05) than those in *Spirodela*, ranging from 36.8% for histidine to 56.9% for arginine, but not different from those in rapeseed meal.

## 4. Discussion

The CP content in soybean meal (511 g/kg DM) was within the reported range (494 to 553 g/kg DM), whereas that in rapeseed meal (335 g/kg DM) was lower than the tabulated values (358 to 415 g/kg DM; [20,21]). The EE content in soybean meal (12 g/kg DM) and rapeseed meal (14 g/kg DM) was also lower, with reported ranges of 14 to 28 g/kg DM and 28 to 43 g/kg DM, respectively. However, the NDF content in soybean meal (107 g/kg DM) and rapeseed meal (269 g/kg DM) was within the reported ranges of 99 to 182 g/kg DM and 30 to 356 g/kg DM, respectively. These differences in chemical composition are likely influenced by various oilseed processing techniques [22], as well as variations in geographical regions, temperature, rainfall, and genetic diversity [23].

The chemical composition of the BSFL observed in the present study was within the ranges reviewed by Barragan-Fonseca et al. [24], with the CP content ranging from 385 to 627 g/kg DM and the EE content ranging from 66 to 392 g/kg DM. However, the nutritional composition of BSFL may vary depending on its life cycle stage and the feeding substrates used [25]. For duckweed, the EE contents in *Lemna* (53 g/kg DM) and *Spirodela* (48 g/kg DM) were consistent with those in previous research, which reported values ranging from 34 to 90 g/kg DM [11]. Similarly, the CF and ash contents in *Lemna* (109 and 142 g/kg DM, respectively) and *Spirodela* (171 and 212 g/kg DM, respectively) were within the ranges reported in the literature, which vary from 88 to 297 g/kg DM for CF content and 35 to 260 g/kg DM for ash content [11,12]. The variation in the chemical composition of duckweed is highly influenced by several factors, including water quality, fertilizer application, cultivation conditions, and duckweed species [3,26,27].

The SID of CP and most AAs obtained in the present study was within the ranges of the values reported for soybean meal and rapeseed meal [20,21], as well as for BSFL [28,29] and duckweed [12]. It should be noted that ileal digesta were collected at 35 days of age, when broilers have a fully developed digestive system with high digestive enzyme activity and absorptive capacity, which may contribute to the high SID values of some AAs (up to 96%) observed in this study. Similarly, SID values of all AAs in corn (up to 100%) were higher in birds at 35 days of age than at 28 days of age at the time of collections [15]. Furthermore, basal endogenous AA losses were estimated using a nitrogen-free diet containing purified cellulose, as proposed by Adedokun et al. [17], which provides a standardized but simplified representation of dietary fiber and may not fully reflect the complex fiber structures present in high-fiber ingredients such as duckweed. This result suggests that the variations in SID values are largely attributed to bird age and the correction for basal endogenous AA losses.

The lower SID of CP and AAs in *Lemna*, *Spirodela*, and rapeseed meal compared to in BSFL and soybean meal likely reflects the influence of tannin, as it has been reported to adversely affect CP and AA digestibility by forming tannin–protein complexes that resist digestion [30]. Additionally, tannin can bind to proteolytic enzymes in poultry, reducing their activity and impairing digestion [31]. This reduction triggers a negative feedback mechanism, increasing the secretion of digestive enzymes and leading to higher endogenous AA losses [32]. Previous studies have reported that a higher tannin content in feedstuffs can reduce AA digestibility in broiler chickens [33]. Consistent with this finding, the high tannin content observed in *Spirodela*, *Lemna*, and rapeseed meal may partly explain their lower AA digestibility than soybean meal and BSFL. Therefore, the tannin content may limit the effectiveness of duckweed as a sustainable feed ingredient.

An increase in NDF and ADF in feed ingredients has been reported to adversely affect AA digestibility due to their abrasive effect on the intestinal wall, which increases endogenous losses [34], as well as the absence of endogenous enzymes capable of breaking down these fiber fractions [35]. Additionally, dietary fiber may interact with nutrients in the digesta, leading to an increased rate of passage through the digestive tract [36]. In the present study, higher NDF and ADF contents were consistently associated with lower AA digestibility in *Spirodela*, *Lemna,* and rapeseed meal compared to in BSFL and soybean meal. It should be noted that the fiber fraction in BSFL may partially include chitin from the insect exoskeleton, which is measured as part of crude fiber but cannot be specifically quantified using conventional fiber analysis [37]. This finding aligns with that of previous research [38], which reported that AA digestibility is generally lower in rapeseed meal than in soybean meal due to its higher fiber content. In contrast, limited information is available on AA digestibility in duckweed, particularly in relation to its fiber content. Notably, the relatively large variation observed for the SID of CP in *Lemna* diets may reflect the compositional variability of duckweed, particularly with respect to fiber fractions and other antinutritional factors, which may contribute to differences in nutrient digestion among birds. Therefore, the results of the present study indicate that the higher NDF and ADF contents in *Spirodela* and *Lemna* may restrict the utilization of duckweed in poultry diets.

The high fat content in feed ingredients may extend the retention time of digesta in the digestive tract, allowing for prolonged protein digestion [39]. Previous research has shown that AA digestibility is influenced by the fat content, with higher digestibility typically observed in high-fat insect meals than in low-fat insect meals [28,29]. This is in agreement with the present study, where the high fat content in BSFL resulted in the SID of CP and most AAs being higher than that in other groups. Notably, the ash content can serve as an indicator of inorganic minerals, which are indigestible compounds that may reduce AA digestibility [40]. An excessive ash content may hinder the absorption of AAs, thereby reducing their digestibility. This finding is supported by the present study, which observed that the high ash content in *Spirodela* and *Lemna* was associated with reduced AA digestibility. However, although BSFL have a higher ash content than soybean meal and rapeseed meal, this ash content is largely associated with the chitin–protein complex of the insect exoskeleton. Interestingly, chickens can produce chitinase in proventriculus and hepatocytes, leading to chitin degradation [41]. Furthermore, the high fat content in BSFL may compensate by extending the digestion time, thereby enhancing AA digestibility [39].

## 5. Conclusions

The high fat content in BSFL can enhance the SID of CP and AA, whereas the use of duckweed may be limited by its fiber fractions and tannin and ash contents, which negatively impact the SID of CP and AAs. However, digestibility may be improved by using higher-quality duckweed with greater CP and AA contents. Therefore, these findings highlight the potential of BSF larvae and duckweed as suitable feed ingredients; however, differences in chemical composition and AA digestibility should be carefully considered during feed formulation to meet the nutritional requirements and effectively optimize the growth performance of broiler chickens.

## Figures and Tables

**Table 1:** Chemical composition of test ingredients (g/kg, as dry matter).

Item	Soybean Meal	Rapeseed Meal	Black Soldier Fly Larvae	*Lemna*	*Spirodela*
Dry matter	930	931	936	945	935
Crude protein	511	335	391	185	145
Ether extract	12	14	95	53	48
Ash	72	109	112	142	212
Crude fiber	30	80	82	109	171
Neutral detergent fiber	107	269	165	316	338
Acid detergent fiber	68	187	90	185	244
Tannin ^1^	1.3	5.7	-	5.5	14.1
Essential amino acids					
Arginine	38.2	24.7	21.7	13.4	11.2
Histidine	13.9	10.3	12.9	3.9	2.9
Isoleucine	25.2	15.2	18.4	9.0	6.6
Leucine	41.1	26.0	29.0	16.4	11.8
Lysine	32.1	17.3	25.5	10.8	8.6
Methionine	6.8	7.1	6.4	3.5	2.5
Phenylalanine	28.1	15.8	16.8	10.4	7.5
Threonine	20.7	15.7	16.7	9.4	7.3
Valine	25.7	19.3	27.4	11.8	9.0
Non-essential amino acids					
Alanine	22.9	16.2	31.0	13.7	11.1
Aspartic acid	60.3	25.1	37.0	22.4	20.5
Glutamic acid	96.8	69.1	47.8	22.5	17.1
Glycine	22.1	19.7	24.3	11.0	8.3
Proline	27.5	24.6	26.6	9.2	6.9
Serine	26.6	15.6	18.7	9.2	7.1
Cystine	2.3	1.4	1.1	0.4	0.4
Tyrosine	20.7	12.0	29.4	7.7	6.1

^1^ Tannin values are expressed as mg/g dry matter, which is numerically equivalent to g/kg dry matter.

**Table 2:** Composition of experimental diets (g/kg, as-fed basis).

Item	Soybean Meal	Rapeseed Meal	Black Soldier Fly Larvae	*Lemna*	*Spirodela*
Soybean meal	404	-	-	-	-
Rapeseed meal	-	473	-	-	-
Black soldier fly larvae	-	-	394	-	-
*Lemna*	-	-	-	535	-
*Spirodela*	-	-	-	-	619
Dextrose	517	448	527	386	302
Dicalcium phosphate	19	19	19	19	19
Soybean oil	40	40	40	40	40
Calcium carbonate	10	10	10	10	10
Salt	2	2	2	2	2
Sodium bicarbonate	2	2	2	2	2
Chromic oxide	3	3	3	3	3
Premix ^1^	3	3	3	3	3

^1^ The premix provided the following per kg of diet: vitamin A 12,000 IU; vitamin D_3_ 1800 IU; vitamin E 12.0 mg; vitamin K_3_ 1.2 mg; vitamin B_1_ 1.2 mg; vitamin B_2_ 6.0 mg; vitamin B_6_ 1.8 mg; vitamin B_12_ 12.0 mg; pantothenic acid 12.0 mg; folic acid 600.0 mg; nicotinic acid 24.0 mg; choline chloride 300.0 mg; Cu 12.0 mg, Zn 96.0 mg; Fe 36.0 mg; Mn 120.0 mg; I 2.4 mg; Co 1.2 mg; Se 1.2 mg; preservative 60 mg.

**Table 3:** Composition of nitrogen-free diet (g/kg, as-fed basis).

Item	Amount
Corn starch	200.5
Dextrose	640.0
Soybean oil	50.0
Cellulose	50.0
Calcium carbonate	13.0
Monocalcium phosphate	19.0
Potassium carbonate	2.6
Magnesium oxide	2.0
Sodium bicarbonate	7.5
Choline chloride	2.5
Potassium chloride	2.9
Chromic oxide	5.0
Premix ^1^	5.0

^1^ The premix provided the following per kg of diet: vitamin A 12,000 IU; vitamin D_3_ 1800 IU; vitamin E 12.0 mg; vitamin K_3_ 1.2 mg; vitamin B_1_ 1.2 mg; vitamin B_2_ 6.0 mg; vitamin B_6_ 1.8 mg; vitamin B_12_ 12.0 mg; pantothenic acid 12.0 mg; folic acid 600.0 mg; nicotinic acid 24.0 mg; choline chloride 300.0 mg; Cu 12.0 mg, Zn 96.0 mg; Fe 36.0 mg; Mn 120.0 mg; I 2.4 mg; Co 1.2 mg; Se 1.2 mg; preservative 60 mg.

**Table 4:** Apparent ileal digestibility of crude protein and amino acids of soybean meal, rapeseed meal, black soldier fly, and duckweed in broiler chickens ^1^.

Item	Soybean Meal	Rapeseed Meal	Black Soldier Fly Larvae	*Lemna*	*Spirodela*	*p*-Value
Crude protein	75.8 ± 2.7 ^a^	64.8 ± 5.3 ^b^	82.5 ± 3.5 ^a^	60.8 ± 11.9 ^b^	35.5 ± 2.8 ^c^	<0.001
Essential amino acids						
Arginine	80.3 ± 1.9 ^b^	75.4 ± 5.1 ^c^	85.3 ± 2.5 ^a^	72.4 ± 5.5 ^c^	47.1 ± 2.6 ^d^	<0.001
Histidine	77.9 ± 3.4 ^b^	70.4 ± 4.6 ^c^	84.1 ± 1.8 ^a^	60.6 ± 7.8 ^d^	19.7 ± 5.3 ^e^	<0.001
Isoleucine	75.1 ± 2.7 ^b^	62.2 ± 5.7 ^c^	82.2 ± 2.6 ^a^	61.9 ± 9.3 ^c^	27.2 ± 4.0 ^d^	<0.001
Leucine	77.0 ± 2.9 ^a^	64.9 ± 5.6 ^b^	83.2 ± 2.3 ^a^	65.1 ± 11.2 ^b^	34.1 ± 3.3 ^c^	<0.001
Lysine	76.7± 3.5 ^b^	63.6 ± 2.3 ^c^	83.5 ± 4.2 ^a^	63.7 ± 8.4 ^c^	31.5 ± 5.0 ^d^	<0.001
Methionine	75.1 ± 2.8 ^ab^	67.0 ± 6.6 ^c^	81.2 ± 3.8 ^a^	68.9 ± 7.0 ^bc^	29.5 ± 4.7 ^d^	<0.001
Phenylalanine	77.3 ± 2.3 ^a^	66.6 ± 5.6 ^b^	81.2 ± 4.1 ^a^	65.7 ± 9.7 ^b^	33.8 ± 3.6 ^c^	<0.001
Threonine	70.2 ± 2.4 ^b^	59.1 ± 4.3 ^c^	76.4 ± 2.4 ^a^	56.3 ± 7.7 ^c^	14.5 ± 4.1 ^d^	<0.001
Valine	73.7 ± 3.3 ^b^	61.2 ± 5.2 ^c^	84.6 ± 1.9 ^a^	63.3 ± 9.9 ^c^	29.2 ± 3.1 ^d^	<0.001
Non-essential amino acids						
Alanine	73.9 ± 3.3 ^b^	63.2 ± 6.4 ^c^	85.8 ± 2.5 ^a^	68.8 ± 5.0 ^b^	45.5 ± 3.0 ^d^	<0.001
Aspartic acid	74.8 ± 1.9 ^b^	63.7 ± 2.5 ^c^	81.8 ± 2.4 ^a^	62.9 ± 4.5 ^c^	53.0 ± 2.3 ^d^	<0.001
Glutamic acid	79.5 ± 1.4 ^a^	72.9 ± 3.8 ^b^	82.7 ± 2.5 ^a^	65.4 ± 7.2 ^c^	30.0 ± 3.5 ^d^	<0.001
Glycine	73.8 ± 2.6 ^b^	62.7 ± 3.9 ^c^	79.2 ± 1.9 ^a^	60.5 ± 6.2 ^c^	28.2 ± 3.3 ^d^	<0.001
Proline	75.5 ± 2.7 ^b^	64.0 ± 3.6 ^c^	86.1 ± 1.7 ^a^	61.2 ± 5.9 ^c^	24.3 ± 3.4 ^d^	<0.001
Serine	75.3 ± 1.9 ^a^	62.1 ± 2.7 ^b^	80.0 ± 2.4 ^a^	54.4 ± 8.5 ^c^	20.2 ± 2.1 ^d^	<0.001
Cystine	74.1 ± 3.4 ^a^	67.4 ± 4.1 ^a^	73.3 ± 3.9 ^a^	34.2 ± 9.1 ^b^	15.5 ± 4.2 ^c^	<0.001
Tyrosine	76.7 ± 2.7 ^b^	63.6 ± 4.1 ^c^	88.3 ± 2.1 ^a^	65.0 ± 8.1 ^c^	25.0 ± 2.6 ^d^	<0.001

^1^ Data are shown as the mean ± standard deviation (SD). Number of replications was 6 (*n* = 6), with three birds per replication. ^a–e^ Within a row, means with the same superscript are not significantly different at *p* < 0.05.

**Table 5:** Standardized ileal digestibility (SID) of crude protein and amino acids of soybean meal, rapeseed meal, black soldier fly, and duckweed in broiler chickens ^1^.

Item	Soybean Meal	Rapeseed Meal	Black Soldier Fly Larvae	*Lemna*	*Spirodela*	*p*-Value
Crude protein	82.3 ± 2.7 ^a^	71.3 ± 5.3 ^b^	89.0 ± 3.5 ^a^	70.2 ± 11.9 ^b^	44.9 ± 2.8 ^c^	<0.001
Essential amino acids						
Arginine	85.0 ± 1.9 ^b^	81.6 ± 5.1 ^bc^	93.7 ± 2.5 ^a^	80.1 ± 2.9 ^c^	56.9 ± 2.6 ^d^	<0.001
Histidine	83.2 ± 3.4 ^b^	76.6 ± 4.6 ^c^	90.0 ± 1.8 ^a^	75.2 ± 7.8 ^c^	36.8 ± 5.3 ^d^	<0.001
Isoleucine	81.1 ± 2.7 ^b^	70.6 ± 5.7 ^c^	90.5 ± 2.6 ^a^	70.6 ± 11.4 ^c^	41.3 ± 4.0 ^d^	<0.001
Leucine	82.7 ± 2.9 ^b^	72.6 ± 5.6 ^c^	91.4 ± 2.3 ^a^	73.3 ± 11.2 ^c^	46.4 ± 3.3 ^d^	<0.001
Lysine	82.4 ± 3.5 ^b^	72.7 ± 2.3 ^c^	90.8 ± 4.2 ^a^	76.3 ± 8.4 ^bc^	45.3 ± 5.0 ^d^	<0.001
Methionine	82.2 ± 4.3 ^b^	74.0 ± 6.6 ^c^	90.4 ± 3.8 ^a^	79.4 ± 7.5 ^bc^	43.0 ± 5.1 ^d^	<0.001
Phenylalanine	82.5 ± 2.3 ^b^	74.6 ± 5.6 ^c^	90.1 ± 4.1 ^a^	74.6 ± 7.9 ^c^	45.7 ± 3.6 ^d^	<0.001
Threonine	79.9 ± 2.4 ^b^	76.6 ± 4.3 ^b^	96.0 ± 2.4 ^a^	73.3 ± 7.5 ^b^	43.3 ± 8.4 ^c^	<0.001
Valine	79.2 ± 4.3 ^b^	70.8 ± 5.8 ^c^	91.2 ± 1.9 ^a^	74.2 ± 9.9 ^bc^	41.4 ± 3.1 ^d^	<0.001
Non-essential amino acids						
Alanine	80.9 ± 3.3 ^b^	71.7 ± 6.4 ^c^	91.1 ± 2.5 ^a^	77.4 ± 5.0 ^b^	54.5 ± 3.0 ^d^	<0.001
Aspartic acid	79.8 ± 1.8 ^b^	73.6 ± 3.3 ^c^	90.6 ± 2.4 ^a^	73.4 ± 4.5 ^c^	61.5 ± 2.6 ^d^	<0.001
Glutamic acid	83.3 ± 1.4 ^b^	77.6 ± 3.8 ^c^	89.4 ± 4.0 ^a^	77.7 ± 7.2 ^c^	42.8 ± 4.3 ^d^	<0.001
Glycine	83.8 ± 3.7 ^b^	72.2 ± 4.6 ^c^	89.7 ± 1.9 ^a^	76.8 ± 7.5 ^c^	46.7 ± 3.3 ^d^	<0.001
Proline	82.0 ± 2.4 ^b^	69.6 ± 3.6 ^d^	92.3 ± 1.7 ^a^	75.4 ± 5.6 ^c^	37.4 ± 4.2 ^e^	<0.001
Serine	86.3 ± 1.9 ^b^	78.3 ± 2.7 ^c^	96.1 ± 2.4 ^a^	75.1 ± 4.5 ^c^	44.5 ± 3.5 ^d^	<0.001
Cystine	80.7 ± 2.7 ^a^	75.3 ± 4.1 ^a^	83.6 ± 5.2 ^a^	44.8 ± 14.0 ^b^	30.4 ± 6.2 ^c^	<0.001
Tyrosine	83.3 ± 2.7 ^b^	72.1 ± 5.0 ^c^	93.0 ± 2.1 ^a^	74.7 ± 10.1 ^c^	39.2 ± 2.6 ^d^	<0.001

^1^ Values for SID were calculated by correcting apparent ileal digestibility values for basal endogenous losses. Basal endogenous losses were determined (g/kg DM intake) as crude protein 13.32; arginine 0.72; histidine, 0.30; isoleucine, 0.61; leucine, 0.94; lysine, 0.74; methionine, 0.23; phenylalanine, 0.59; threonine, 0.80; valine, 0.71; alanine, 0.65; aspartic acid, 1.29; glutamic acid, 1.52; glycine, 0.67; proline, 0.65; serine, 0.70; cystine, 0.50; tyrosine, 0.55. Data are shown as the mean ± standard deviation (SD). Number of replications was 6 (*n* = 6), with three birds per replication. ^a–e^ Within a row, means with the same superscript are not significantly different at *p* < 0.05.

## Data Availability

The data presented in this study are available on request from the corresponding author.
